# Physics-Guided Descriptors for Prediction of Structural Polymorphs

**DOI:** 10.1021/acs.jpclett.2c01876

**Published:** 2022-08-03

**Authors:** Bastien F. Grosso, Nicola A. Spaldin, Aria Mansouri Tehrani

**Affiliations:** Materials Theory, ETH Zürich, Wolfgang-Pauli-Strasse 27, 8093 Zürich, Switzerland

## Abstract

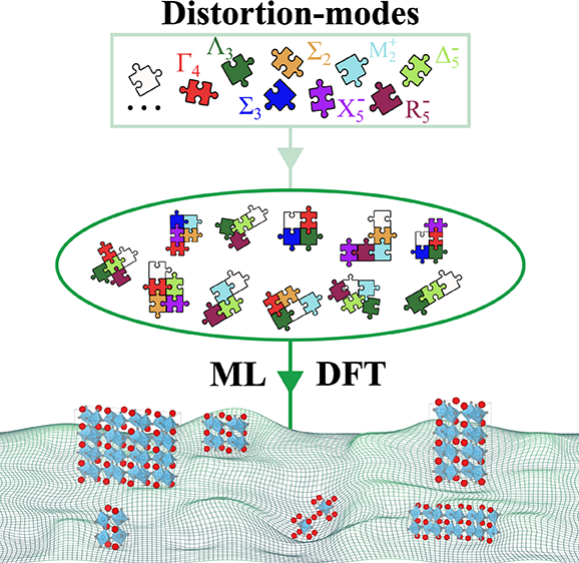

We develop a method combining machine learning (ML) and density functional theory (DFT) to predict low-energy polymorphs by introducing physics-guided descriptors based on structural distortion modes. We systematically generate crystal structures utilizing the distortion modes and compute their energies with single-point DFT calculations. We then train a ML model to identify low-energy configurations on the material’s high-dimensional potential energy surface. Here, we use BiFeO_3_ as a case study and explore its phase space by tuning the amplitudes of linear combinations of a finite set of distinct distortion modes. Our procedure is validated by rediscovering several known metastable phases of BiFeO_3_ with complex crystal structures, and its efficiency is proved by identifying 21 new low-energy polymorphs. This approach proposes a new avenue toward accelerating the prediction of low-energy polymorphs in solid-state materials.

Computational materials science has undergone a recent paradigm shift with the advent of data-driven methods such as machine learning (ML). These techniques are now considered a standard tool, along with widely used methods such as density functional theory (DFT), molecular dynamics, or Monte Carlo simulations.

One central task for effectively applying machine learning algorithms to materials science problems is developing appropriate descriptors. Descriptors are vector-based numerical representations that should uniquely define the material and are mainly based on compositional or structural features or a mixture of both. In addition, these descriptors should provide meaningful connections to the physics of the materials by establishing a unique and invariant “barcode” for each one of them.

The advantage of descriptors based only on the chemical composition, such as atomic number, covalent radius, and number of valence electrons,^1^ is that no prior knowledge of the system is required. However, while this approach has been successful in predicting different properties such as band gap, hardness, and thermodynamic stability,^2−5^ it is not capable of distinguishing between different structures of the same composition (polymorphs). This problem is highly important because, while metastable in bulk, structural polymorphs can in many cases be stabilized through strain, pressure, or electrostatic boundary conditions and often exhibit new properties absent in the bulk ground state enabling new applications.^6,7^ It is therefore crucial to incorporate the influence of structural features to correctly describe phenomena such as metal–insulator transitions or ferroelectricity among others.^8−10^ Descriptors based on structural features, such as the smooth overlap of atomic positions (SOAP),^11^ minimum bounding ellipsoid (MBE),^12^ ordered eigenvalues of the Coulomb matrix,^13^ or universal fragment descriptors,^14^ have been developed to capture the local environment of the atoms and encode structural features.

A natural approach to representing a crystal structure is to use the concept of irreducible representations (irreps), in which the distortions from a higher symmetry reference structure are decomposed into a set of normal modes that describe the transformation of the reference structure into the considered one.^15,16^ This approach has been used to understand and explain various phenomena such as thermopower anisotropy, negative thermal expansion, proper and improper ferroelectricity, and antiferroelectricity.^17−21^ Furthermore, in the context of phase transitions in transition-metal compounds, the distortion modes have been used as features in statistical analysis of the correlation between distortions and functionalities.^8,22,23^ More recently, polyhedral distortions have been used as descriptors to explain trends in behaviors across perovskites.^24^

In this work, we explore the idea of using the distortion modes as descriptors in ML. Our method utilizes distortion modes to explore the Born–Oppenheimer potential energy surface (PES) and identify local minima corresponding to metastable phases (polymorphs). While the method that we introduce remains applicable to any crystalline material, here we focus on perovskites for which the distortion modes are simply obtained as a decomposition of the high-symmetry prototype perovskite parent structure with *Pm*3̅*m* symmetry. We apply the method to multiferroic bismuth ferrite, BiFeO_3_ (BFO), which exhibits simultaneous ferroelectricity and magnetic ordering and has a rich structural landscape composed of many local minima with interesting technological properties.^25,26^

The ground state structure of BFO (*R*3*c*) has a 10-atom unit cell and is reached from the *Pm*3̅*m* prototype by a combination of polar displacement of the Bi cation (mode at Γ) and antiphase rotation of consecutive oxygen octahedra in all three Cartesian directions (mode at *R*).^27^ Several metastable phases have been established computationally, many with larger unit cells.^26^ Furthermore, the experimental stabilization of many new phases of BFO^28−30^ motivates our choice of BFO both to test the predictive power of the method and to identify new metastable phases for future experimental study.

When a structure experiences a symmetry lowering, part of its symmetry elements are lost, and the remaining symmetry operations constitute a subgroup of the initial parent space group. If there exists a group–subgroup relation between the structures, one can decompose the distorted one into symmetry-adapted modes of the parent structure that encode patterns of displacements of the atoms. For example, the perfect perovskite structure with space group *Pm*3̅*m* can be considered as the higher symmetry parent structure of all lower symmetry structures that are distorted versions of the *Pm*3̅*m* phase. It becomes then clear that different subgroups of *Pm*3̅*m* can share common symmetry modes. Nevertheless, each structure is characterized by a unique combination of symmetry modes with given relative amplitudes.

As each mode consists of a displacement pattern of the atoms, the combination of different modes can either lower or increase the structure’s total energy compared to the undistorted perovskite structure. The increase in energy can occur, for example, in the case of the interatomic distances between anions and cations becoming too small. While chemical intuition could, in principle, allow one to create simple subgroups of distortion modes, resulting in (meta)stable structures, the number of possibilities and the complexity of many distortions are prohibitively large, and a more systematic approach, which we propose here, is required.

Our study starts with selecting the distortion modes that will form our descriptors. To select appropriate distortion modes, we extract from the phases previously reported in refs (25) and (26) all the different modes that are contained in unit cells of 80 atoms or fewer using ISODISTORT.^15,16^ This yields the 15 modes shown in Figure 1a. Because certain modes result in displacements that can arbitrarily be applied along different directions, we include six additional building block structures obtained by linear combinations of the nonrotational distortion modes along the directions defined by the lattice vectors. We show an example in Figure 1b with the X_5_^–^ mode, where we take the linear combination of the structures obtained by this mode applied along two different directions (**a** and **b**) and obtain a structure where the distortions are along **ab**. While the structures with the distortions along **a** and **b** are identical, the new structure with the distortions along the diagonal of the unit cell has a different symmetry and can then be considered as a building block, keeping the uniqueness of the descriptors. We then obtained 21 building blocks constructed by distorting a perfect cubic supercell according to 15 different modes and six of their spatial variations.

**Figure 1:**
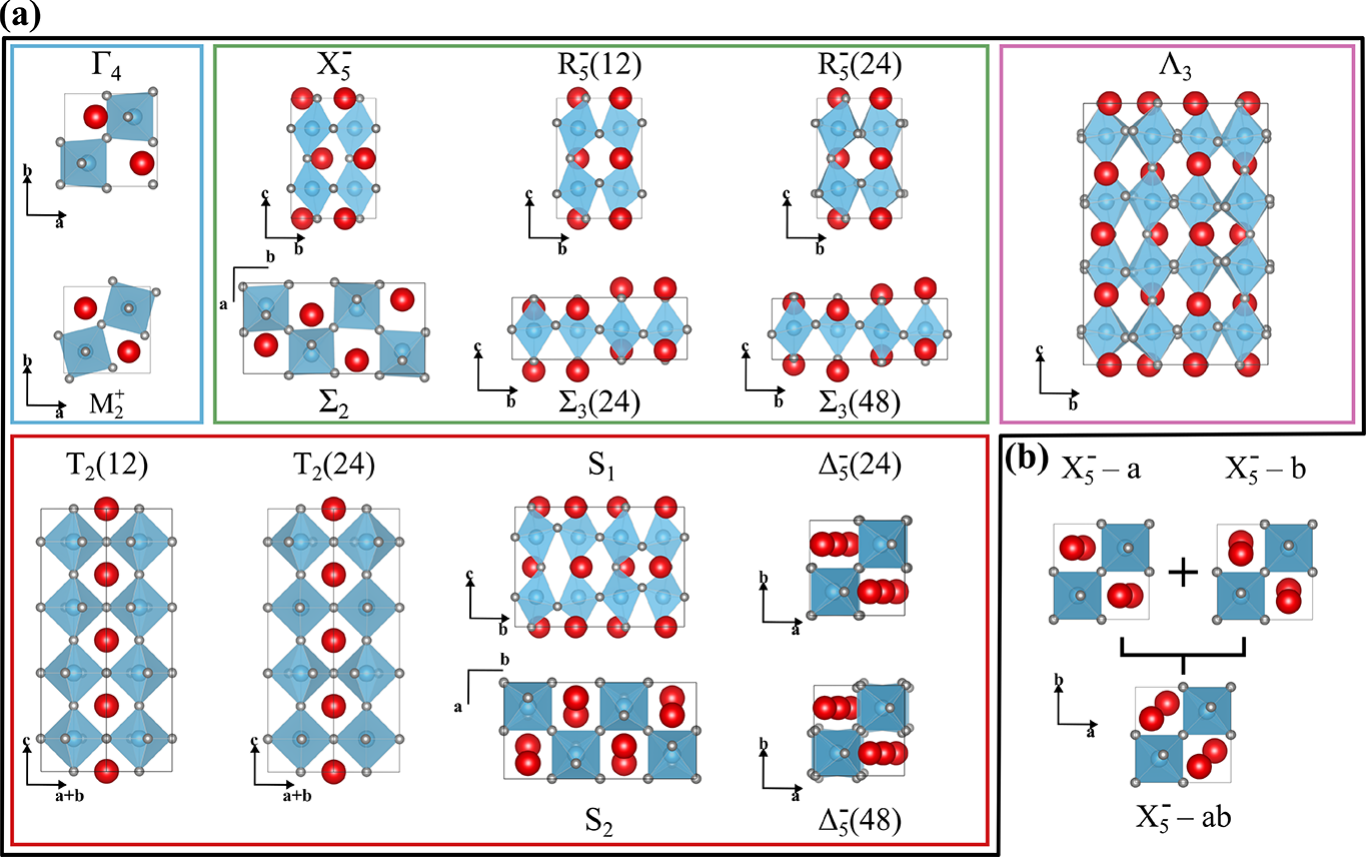
(a) Structures of the different distortion modes used as descriptors. The structures are gathered according to the number of atoms each primitive unit cell contains and contain 10, 20, 40, and 80 atoms per unit cell for the blue, green, red, and pink boxes, respectively. Note that we distinguish between distortion modes with the same symmetry by indicating the corresponding symmetry index in parentheses. (b) Structure of a distortion mode along orthogonal directions illustrates how we create extra descriptors with different orientations. Because the structures corresponding to the distortions imposed by the X_5_^–^ mode along **a** and **b** are identical, we include in our set of building blocks the symmetrically distinct structures obtained from X_5_^–^–*a* the X_5_^–^–*ab*. The remaining structures are shown in Figure S1.

Using symmetry considerations, we reduce the unit cell size of the distorted structure to its minimal possible size. We then fix its volume to match that of a supercell of the *Pm*3̅*m* structure (Figure 2a) coinciding with the distorted structure. This constrains the structure to four possible unit cell sizes displayed in Figure 2b, all of which accommodate G-type antiferromagnetic order. Furthermore, we normalize all the modes in such a way that the sum of the displacements multiplied by  is equal to 1 Å, equivalent to a mode amplitude of 1, where *V*_p_ and *V*_s_ are the primitive and supercell volumes, respectively. This allows us to describe evenly all modes, no matter the size of their unit cell.

**Figure 2:**
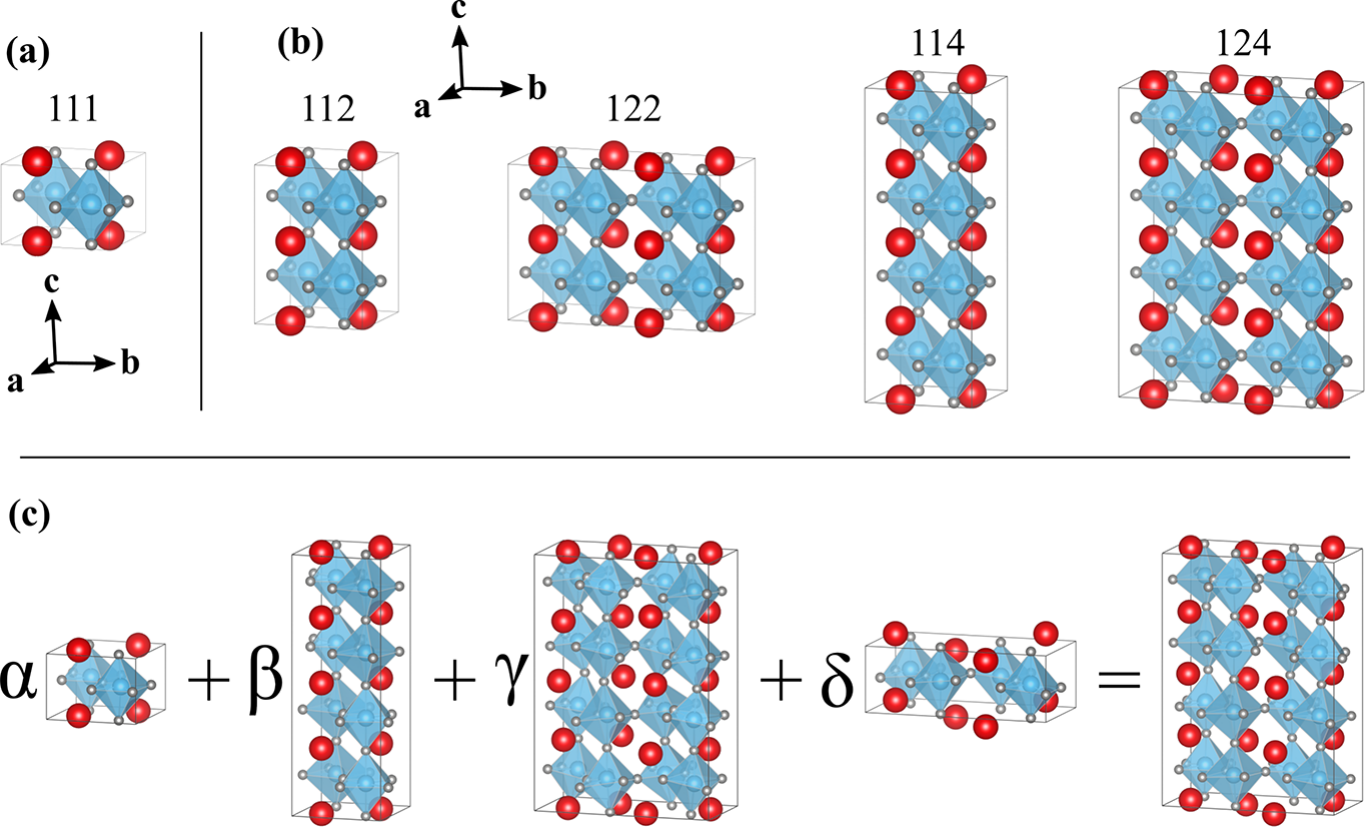
(a) Basic cubic unit cell labeled 111. (b) Possible supercells. Each supercell is constructed from multiples of the cubic unit cell structure in (a) along each Cartesian direction and labeled accordingly. (c) Example of a combination of four modes to build a structure. All structures of the modes are scaled to the size of the structure of the third mode (which has the largest supercell), and for each mode, we create a distortion vector capturing the displacement from the 124 structure displayed in (b) and the considered mode. Each vector is then multiplied by the corresponding amplitude factor (α, β, γ, or δ), and the vectors are summed to give the final structure.

Finally, a structure can be created by displacing the atoms in the parent structure by a sum of displacements resulting from each normalized mode multiplied by a scalar amplitude, as represented in Figure 2c. The displacements are computed in supercells corresponding to the largest structure present in the set of modes selected (see Figure 2b).

In Figure 3, we show our combined DFT and ML approach, which proceeds in two rounds. In the first round, the model is trained by building a training set on distortion modes and computing the energies of the structures by using DFT single-point calculations. In this stage, only the electrons are relaxed while the ions are kept fixed to determine the energies as a function of the modes’ amplitudes. In the second round, using the same methodology, we generate a large number of structures and utilize our ML model, instead of the more expensive DFT calculations, to predict their energies. Finally, we perform DFT ionic relaxation for a selected number of low-energy structures. This approach allows us to start the DFT relaxation on initial structures predicted to be low energy by our ML model and thus are likely to be close to a local minimum of the potential energy surface.

**Figure 3:**
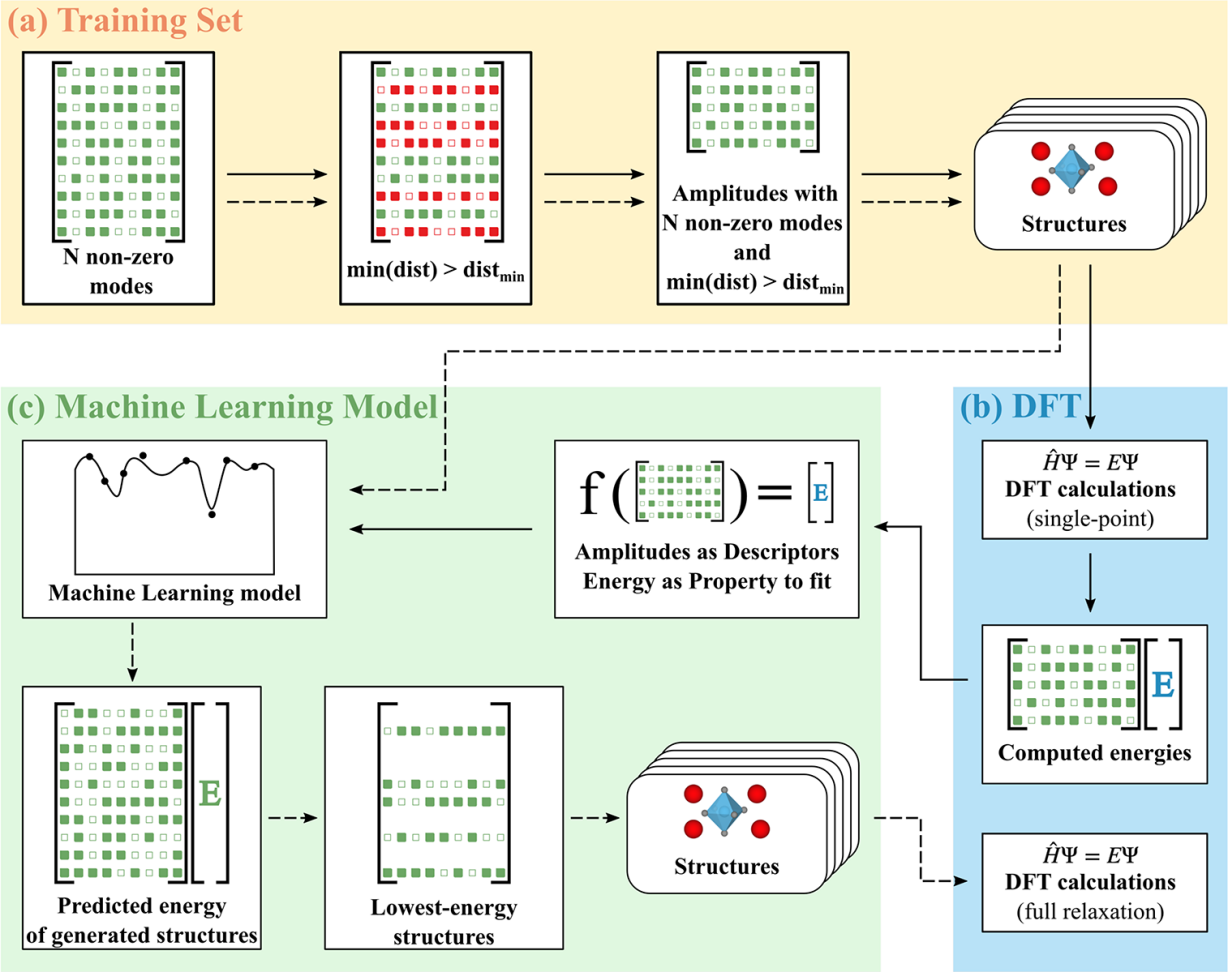
Overview of our DFT-ML procedure. The first round is indicated by solid arrows and the second by dashed ones. (a) (yellow) Construction of the training set. For a given value of *N*, we start from a list of *N* nonzero random amplitudes (first panel), where each row of the list corresponds to a structure and each column to the amplitude of a given distortion mode. Nonzero amplitudes are represented by full squares and zero amplitudes by empty squares. From the list, we discard (red) the structures in which two atoms are closer than the minimum distance allowed (dist_min_) (second and third panels). Finally, we generate the input files corresponding to the structures (fourth panel) that we retain for the DFT section. (b) (blue) DFT calculations for electronic relaxation (b, top) or electronic, ionic, and volumetric relaxation (b, bottom). We compute the energy with a single-point DFT calculation for each line of the list. (c) Using ML, we use the list of amplitudes gathered in the previous step as descriptors and fit the corresponding energies. We then obtain a model that can predict the energy of a given combination of amplitudes. Dashed arrows represent the second round. Following the same steps as described in the first round, we (a) generate structures with different values of *N*. Then, we use the ML model created to predict the energy of those structures, select the lowest energy ones, and create the corresponding structures (c). Finally, we use these structures as starting configurations and fully relax the ions, lattice parameters, and electrons (b).

The first round starts with the generation of the training set. We consider the potential energy surface of BFO in a 21-dimensional space where each dimension is given by a distortion mode, and each coordinate in the space is given by the amplitude of the modes. To span the energy surface unbiasedly, we randomly generate the amplitudes of the modes in the interval [−1.2; 1.2]. This approach generates amplitudes corresponding to the sum of the atomic displacements from the parent structure. The choice of the [−1.2; 1.2] range, even though arbitrary, is set to capture a wide range of the potential energy for each mode. Furthermore, to account for the physics of each mode individually and to capture any favorable or unfavorable couplings up to the seventh order between different modes, we include structures generated by including up to seven modes with amplitudes different than zero.

Our procedure starts (Figure 3a) by choosing an order of coupling *N*, equal to the number of nonzero amplitudes, and generating a matrix of *L* lines and 21 random numbers in the interval [−1.2; 1.2] for each line, where *L* is the number of potential structures. We then obtain a matrix of dimensions *L* × 21. Second, we randomly set amplitude entries per line to zero (21 – *N*). We then discard nonphysical structures, determined by computing the minimum distance between pairs of atoms in each structure and removing the structures for which any minimum distance is too small. On the basis of the distribution of the Fe–O bond lengths in all structures containing these ions in the ICSD database,^31^ we keep only structures with the minimum distance between any pair of atoms larger than 1.8 Å. Note that the Fe–O bond lengths was chosen because it is usually the smallest distance in regular BFO. This procedure is repeated for each value of *N* considered and results in a set of structures ready for the following step. Note that all structures’ volumes are fixed to integer multiples of the cubic undistorted cell.

We then take all the created structures and compute their energies with a single-point DFT calculation in which only one electronic loop is computed. As a result, we obtain the energy for each structure described by a row of amplitudes in the matrix (Figure 3b, top).

We present in Figure S2 the energies of the different structures included in the training set as well as the number of structures per mode. As shown in Figure S2, most of the structures included in the training set have energy lying between that of the *R*3*c* phase constrained to the cubic parent structure volume and angles and that of the cubic parent structure. Furthermore, when the number of modes present in the structure increases, the energy tends also to increase. This is a result of our choice of random amplitudes, for which additional distortions reduce the likely distance between ions and consequently increase the energy.

The last step of the first round (see Figure 3c) consists of using the training set to fit the energies as a function of the modes’ amplitudes. We use a Support Vector Regression (SVR) algorithm, which outperformed other machine learning algorithms that we tried, such as Random Forest and Extreme Gradient Boost (XGB), to construct a model evaluated on the test set. Figure 4a illustrates the reasonable agreement between the machine learning predicted total energies and DFT calculations. The coefficient of determination (*r*^2^) is around 0.77, and the root-mean-square error (RMSE) is determined to be 0.2 eV/f.u. It is worth noting that within the range of energy contained by the constrained *R*3*c* and parent cubic structures (lower left of the plot) the points are less spread around the diagonal line (ML = DFT) than they are in the upper right of the plot. This can be attributed to the lower representation of high-energy structures in the training set.

**Figure 4:**
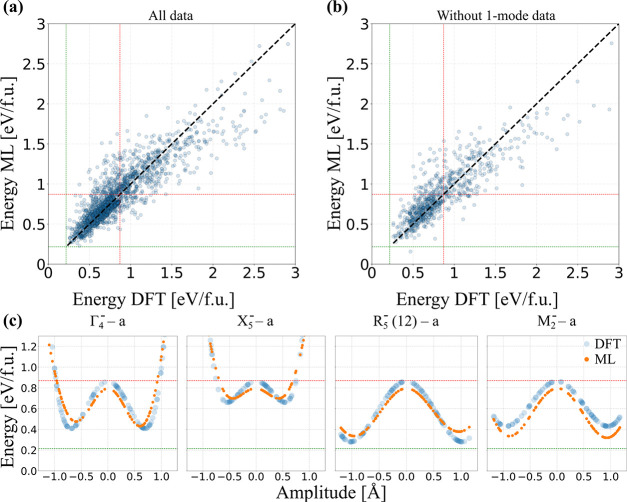
Evaluation of the prediction accuracy of our ML models. (a) Regression plot of predicted (ML) energies versus computed (DFT) energies on the test set. (b) Predicted energy versus computed energy when all the structures containing only one mode were removed from the training set. Each blue dot represents the energy of a structure. The black dashed line represents the position of perfect predictions, where the energy predicted by the ML model perfectly agrees with the DFT energy. The red and green lines are the energy of the cubic and *R*3*c*-like structures, respectively. (c) Computed energies (blue) compared to the energies predicted with the model fitted on the data without the one-mode structures (orange) for four often discussed modes. The red and green lines have the same meaning as in (a) and (b).

Next, we use the permutation feature importance technique to evaluate which features (distortion modes) significantly contribute to our SVR model prediction of the test set. In this technique, we randomly shuffle the value of each feature 30 times and evaluate the variation in the *r*^2^ metric. A feature is deemed important if the shuffling leads to substantial changes in the model’s error. Figure S3 shows the permutation importance of each feature based on changes in *r*^2^ sorted from highest to lowest. On the basis of this analysis, the X_5_^–^ mode along **ab** is the most important feature, and as expected, the Γ_4_^–^ modes are also significant. However, surprisingly, modes corresponding to 40-atom supercells, such as T_2_(24) or Δ_5_^–^(24), are also crucial for the model to predict the energy of various structures, demonstrating the importance of including these less often discussed modes.

We further evaluate the extrapolative power of our model by removing all the structures that contain only one distortion mode (1922 points) from the training set before predicting their energies. Figure 4b shows the predicted energy values by using the SVR model against the DFT energies, constructed on the training set without single distortion modes. As expected, the *r*^2^ evaluated on the test set is now lower (≈74%) with RMSE = 0.21 eV/f.u. compared to the full test set.

Next, utilizing the model built without the single distortion modes, we test whether our machine learning model accurately reproduces the energy variation of the distortion modes with respect to the amplitude by comparing the ML energies to their corresponding DFT energies. Our results are shown in Figure 4c. We see that the SVR model correctly predicts the signature double-well behavior for the Γ_4_^–^–*a*, X_5_^–^–*a*, R_5_^–^(12)–*a*, and M_2_^–^–*a* modes as well as determining the high energy barrier between the two minima for R_5_^–^(12)–*a* and M_2_^–^–*a*. These curves are also plotted for the rest of the individual distortion modes in Figure S4, where we see that good agreement between DFT and ML predictions is obtained for most of them. Thus, despite showing imperfect statistical metrics on the test set, the ML model successfully captures the structural energetics of BFO.

In the second round, we use the constructed model to predict the energies of a large number of structures built with the random composition of 1–7 modes combined. First, we construct 457081 structures in the same way as described earlier and as shown schematically in Figure 3a and predict their energies. Using the model, we select the structures within 250 meV/f.u. of the ground state and classify them into subsets according to their distortion modes. Finally, we select the lowest energy structure of each subset. As a result, we obtain 290 structures, all with different combinations of the modes and containing 20, 40, or 80 atoms, which we then fully relax with DFT.

Focusing only on the structures displaying an energy of 100 meV/f.u. or less above the *R*3*c* ground state after full relaxation, we obtain 29 different structures, among which eight are rediscovered phases: four phases are the common *R*3*c*, *Pnma*, *Pbam*, and *Pmc*2_1_^25,32,33^ and four were reported in ref (26) and labeled *Pc*(1), *Pnma*(1), *Cmc*2_1_(2), and *P*2_1_/*c*. We report the 21 new phases in Table S1. We observe that the majority of the phases found have 80 atoms per unit cell and have an energy around 40–50 meV/f.u. above the ground state. While this is the largest unit cell size allowed by our way of building the structures (see Figure 2b), it suggests that even larger unit cell could display stable phases.

While a full analysis of all these phases are out of the scope of this work, we provide information about their structures in Table S1 and focus on the analysis of the lowest-energy phase found with *P*2_1_ symmetry and present its crystal structure in Figure 5. Note that all the crystal structures are available at the open-source GitHub provided at https://github.com/grossoba/DistortionModesDescriptors. Its unit cell, as shown in Figure 5a, is similar to the Pc(1) phase referenced in ref (26), and it has the same energy difference with respect to the ground state (23 meV/f.u.). While in the Pc(1) structure the Bi cations are displaced perpendicular to the long axis, with three cations moving in one direction and one in the opposite direction, in the *P*2_1_ structure, two Bi cations move in one direction and the next two in the opposite direction (see Figure 5b). The *P*2_1_ structure also has a different rotation pattern with *a*^α̅β^*a*^α̅β^*c*^α̅βγδ̅^ rotations, as opposed to the *a*^α̅β^*a*^α̅β^*c*^α̅βγδ̅^ rotations in Pc(1) (notation adopted from ref (26)). Considering the isotropic Born effective charges of 4.86 [*e*] for Bi, 3.99 [*e*] for Fe, and −2.95 [*e*] for O in units of the electronic charge magnitude, and multiplying the atom displacements with respect to the cubic parent structure by the corresponding isotropic charge,^26^ we evaluate the spontaneous polarization to be around 54 μC/cm^2^ along the *c*-axis (long axis). In Figure 5c we present views of the structures along the pseudocubic orientations and the structures that could be experimentally observed by using high-resolution transmission electron microscopy. In particular, we see that for the view down the *c*-axis in-plane a “2up–2down” displacement pattern of the Bi cations appears along the [100] direction, reminiscent of the recently discovered antiferroelectric *Pnma* phase.^29^

**Figure 5:**
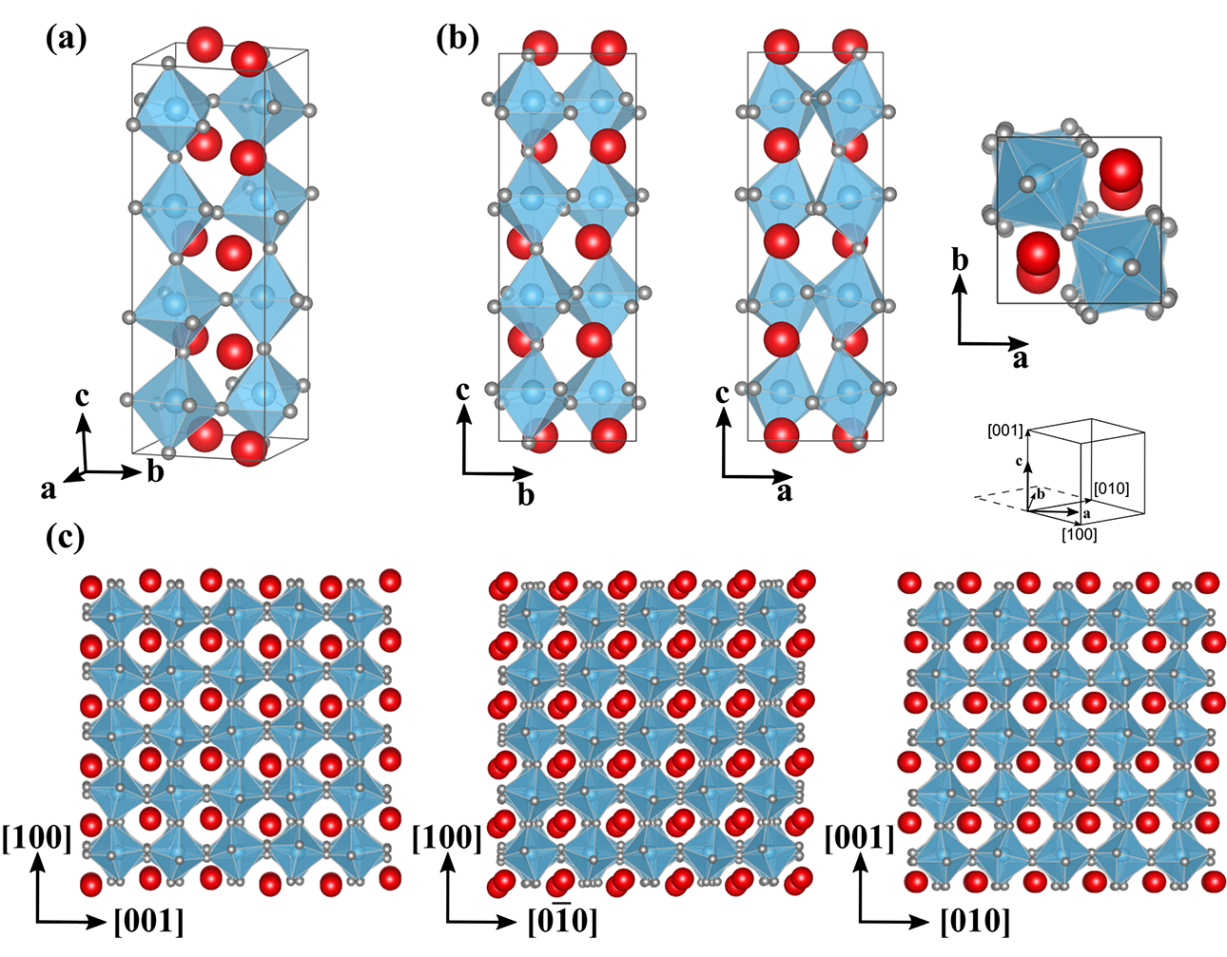
Crystal structure of the *P*2_1_ phase. (a) Full unit cell. (b) *b*–*c*, *a*–*c*, and *a*–*b* projections of the unit cell. (c) *P*2_1_ supercells imaged along the three pseudocubic directions.

In summary, we introduced a new DFT-ML approach to efficiently explore the energy landscape of complex solid-state materials by implementing distortion modes as descriptors. Our approach was successfully implemented within the BiFeO_3_ phase space, rapidly rediscovering low-energy crystal structures that had previously been identified by using various computational and experimental methods. In addition, we predicted 21 new low-energy polymorphs of BFO (<100 meV/f.u. above the ground state), including one of the lowest-energy polymorphs of BFO reported, with the *P*2_1_ symmetry and showing a large polarization. Our predictions (all crystallographic information of the predicitons can be accessed through the GitHub link provided at https://github.com/grossoba/DistortionModesDescriptors) further highlight the rich phase space of BFO and hopefully motivates additional experimental work to synthesize these predictions.

Our approach of utilizing distortion modes as building blocks provides a facile way to generate crystal structures and navigate the energy landscape by controlling the coefficients of their amplitudes. Additionally, while we demonstrated the relevance of the use of distortion modes as descriptors through the search for new metastable phases in BFO, our framework can easily be adapted to other materials and could be used for example in materials undergoing multiple polymorph transitions such as metallic halide perovskites. Moreover, our method could provide significant insights for many other applications. One example is Landau theory for phase transitions.^34^ To elaborate, building a Landau model to study phase transitions in materials involves the study of couplings between distortion modes, which is often limited to second or third order^21^ due to the high computational cost. Our model implicitly contains these couplings, which could readily be extracted at minimal computational cost if one would beforehand relax the volume of the structures (in the training set) to avoid the presence of resulting stress contributions to the energy.

Another potential application of our methodology is in the field of ML-driven interatomic potentials with a focus on crystal structure prediction.^35^ Constructing machine-learning-guided interatomic potentials involves creating an efficient training set of structures. While a random sampling of the phase space is a valid strategy and has presented great success in elemental systems,^36^ we believe that our approach can be a useful complement to efficiently exploring phase spaces and predicting energetically accessible polymorphs.

Finally, while the building blocks of the modes in our example are the atomic displacements, the use of group–subgroup relations and the corresponding irreducible representations as descriptors in machine learning should be more widely applicable. For example, symmetry-allowed combinations of magnetic moments could be used as a basis to search for low-energy magnetic orderings in complex antiferromagnetic systems.

## Computational Details

The DFT calculations are performed by using VASP^37−40^ with the PAW method^41,42^ and explicit treatment of the following valence electrons: 6s^2^6p^3^ for Bi, 3d^7^4s^1^ for Fe, and 2s^2^ 2p^4^ for O. A 8 × 8 × 6 *k*-point Γ-centered Monkhorst–Pack mesh^43^ is used to sample the Brillouin zone of a 20-atom unit cell, and an energy cutoff of 700 eV for the plane-wave basis is chosen. For the xcs functional we use PBEsol^44^ with an effective Hubbard-like correction, *U*_eff_ = 4 eV, for the Fe d orbitals according to Dudarev’s approach.^45^ G-type antiferromagnetism is adopted for all the calculations.

The machine learning model is created based on a Support Vector Machine Regression (SVR) method^46,47^ as implemented in the Scikit-learn^48^ library. We use a SVR model with a radial basis function (RBF) as the kernel function. Using the approach illustrated in Figure 1, we generate 9569 structures and energies using DFT (see Sec. and split the data into a training set (80%, 7655 data points) and a test set (20%, 1914 data points). We further perform a 5-fold cross-validation scheme to optimize the cost constant (*C*) and the RBF free parameter (γ) to be *C* = 10 and γ = 1 for the best performance. We finally use these parameters to evaluate the model on the test set.

## Supporting Information Available

Figures S1–S4 and Table S1 (PDF)
Transparent Peer Review report available (PDF)

## Notes

Training set data, the codes developed to produce the current work, and the structures of the newly identified polymorphs are available in the open-source GitHub repository at https://github.com/grossoba/DistortionModesDescriptors.

## References

[ref1] Zhuo Y.; Mansouri Tehrani A.; Brgoch J. Predicting the Band Gaps of Inorganic Solids by Machine Learning. J. Phys. Chem. Lett. 2018, 9, 1668–1673. 10.1021/acs.jpclett.8b00124. 29532658

[ref2] de Jong M.; Chen W.; Notestine R.; Persson K.; Ceder G.; Jain A.; Asta M.; Gamst A. A Statistical Learning Framework for Materials Science: Application to Elastic Moduli of k-nary Inorganic Polycrystalline Compounds. Sci. Rep. 2016, 6, 34256 10.1038/srep34256. 27694824 PMC5046120

[ref3] Li W.; Jacobs R.; Morgan D. Predicting the Thermodynamic Stability of Perovskite Oxides Using Machine Learning Models. Comput. Mater. Sci. 2018, 150, 454–463. 10.1016/j.commatsci.2018.04.033. PMC5992996 29892644

[ref4] Zhang Z.; Mansouri Tehrani A.; Oliynyk A. O.; Day B.; Brgoch J. Finding the Next Superhard Material through Ensemble Learning. Adv. Mater. 2021, 33, 2005112 10.1002/adma.202005112. 33274804

[ref5] Wang A. Y.-T.; Kauwe S. K.; Murdock R. J.; Sparks T. D. Compositionally Restricted Attention-Based Network for Materials Property Predictions. Npj Comput. Mater. 2021, 7, 77 10.1038/s41524-021-00545-1.

[ref6] Sun W.; Dacek S. T.; Ong S. P.; Hautier G.; Jain A.; Richards W. D.; Gamst A. C.; Persson K. A.; Ceder G. The Thermodynamic Scale of Inorganic Crystalline Metastability. Sci. Adv. 2016, 10.1126/sciadv.1600225. PMC5262468 28138514

[ref7] Khalyavin D. D.; Salak A. N.; Olekhnovich N. M.; Pushkarev A. V.; Radyush Y. V.; Manuel P.; Raevski I. P.; Zheludkevich M. L.; Ferreira M. G. S. Polar and Antipolar Polymorphs of Metastable Perovskite BiFe0.5Sc0.5O3. Phys. Rev. B 2014, 89, 174414 10.1103/PhysRevB.89.174414.

[ref8] Balachandran P. V.; Rondinelli J. M. Interplay of Octahedral Rotations and Breathing Distortions in Charge-Ordering Perovskite Oxides. Phys. Rev. B 2013, 88, 054101 10.1103/PhysRevB.88.054101.

[ref9] He J.; Li J.; Liu C.; Wang C.; Zhang Y.; Wen C.; Xue D.; Cao J.; Su Y.; Qiao L.; Bai Y. Machine Learning Identified Materials Descriptors for Ferroelectricity. Acta Mater. 2021, 209, 116815 10.1016/j.actamat.2021.116815.

[ref10] Frey R.; Grosso B. F.; Fandré P.; Mächler B.; Spaldin N. A.; Masouri Tehrani A. Accelerated Search for New Ferroelectric Materials. ArXiv.2201.05668 2022.

[ref11] Bartók A. P.; De S.; Poelking C.; Bernstein N.; Kermode J. R.; Csányi G.; Ceriotti M. Machine Learning Unifies the Modeling of Materials and Molecules. Sci. Adv. 2017, 10.1126/sciadv.1701816. PMC5729016 29242828

[ref12] Cumby J.; Attfield J. P. Ellipsoidal Analysis of Coordination Polyhedra. Nat. Commun. 2017, 8, 14235 10.1038/ncomms14235. 28146146 PMC5296646

[ref13] Rupp M.; Tkatchenko A.; Müller K.-R.; von Lilienfeld O. A. Fast and Accurate Modeling of Molecular Atomization Energies with Machine Learning. Phys. Rev. Lett. 2012, 108, 058301 10.1103/PhysRevLett.108.058301. 22400967

[ref14] Isayev O.; Oses C.; Toher C.; Gossett E.; Curtarolo S.; Tropsha A. Universal Fragment Descriptors for Predicting Properties of Inorganic Crystals. Nat. Commun. 2017, 8, 15679 10.1038/ncomms15679. 28580961 PMC5465371

[ref15] Stokes T.; Hatch D. M.; Campbell B. J. ISODISTORT, ISOTROPY Software Suite. iso.byu.edu, 2022.

[ref16] Campbell B. J.; Stokes H. T.; Tanner D. E.; Hatch D. M. ISODISPLACE: a Web-Based Tool for Exploring Structural Distortions. J. Appl. Crystallogr. 2006, 39, 607–614. 10.1107/S0021889806014075.

[ref17] Puggioni D.; Rondinelli J. M. Designing a Robustly Metallic Noncenstrosymmetric Ruthenate Oxide with Large Thermopower Anisotropy. Nat. Commun. 2014, 5, 3432 10.1038/ncomms4432. 24633396

[ref18] Senn M.; Bombardi A.; Murray C.; Vecchini C.; Scherillo A.; Luo X.; Cheong S. Negative Thermal Expansion in Hybrid Improper Ferroelectric Ruddlesden-Popper Perovskites by Symmetry Trapping. Phys. Rev. Lett. 2015, 114, 035701 10.1103/PhysRevLett.114.035701. 25659007

[ref19] Nowadnick E. A.; Fennie C. J. Domains and Ferroelectric Switching Pathways in Ca3Ti2O7 from First Principles. Phys. Rev. B 2016, 94, 104105 10.1103/PhysRevB.94.104105.

[ref20] Boström H. L. B.; Senn M. S.; Goodwin A. L. Recipes for Improper Ferroelectricity in Molecular Perovskites. Nat. Commun. 2018, 9, 2380 10.1038/s41467-018-04764-x. 29915202 PMC6006342

[ref21] Shapovalov K.; Stengel M. Tilt-Driven Antiferroelectricity in PbZrO3. arXiv 2021.

[ref22] Balachandran P. V.; Benedek N. A.; Rondinelli J. M. Information Science for Materials Discovery and Design; Springer International Publishing: 2015; pp 213–222.

[ref23] Wagner N.; Puggioni D.; Rondinelli J. M. Learning from Correlations Based on Local Structure: Rare-Earth Nickelates Revisited. J. Chem. Inf. Model 2018, 58, 2491–2501. 10.1021/acs.jcim.8b00411. 30111111

[ref24] Morita K.; Davies D. W.; Butler K. T.; Walsh A. Breaking the Aristotype: Featurization of Polyhedral Distortions in Perovskite Crystals. Chem. Mater. 2022, 34, 562–573. 10.1021/acs.chemmater.1c02959.

[ref25] Dieguez O.; Gonzalez-Vazquez O. E.; Wojdeł J. C.; Iniguez J. First-Principles Predictions of Low-Energy Phases of Multiferroic BiFeO_3_. Phys. Rev. B 2011, 83, 094105 10.1103/PhysRevB.83.094105.

[ref26] Grosso B. F.; Spaldin N. A. Prediction of Low-Energy Phases of BiFeO3 with Large Unit Cells and Complex Tilts Beyond Glazer Notation. Phys. Rev. Mater. 2021, 5, 054403 10.1103/PhysRevMaterials.5.054403.

[ref27] Kubel F.; Schmid H. Structure of Ferroelectric and Ferroelastic Monodomain Crystal of the Perovskite BiFeO_3_. Acta Crystallogr. 1990, B46, 698–702. 10.1107/S0108768190006887.

[ref28] Nordlander J.; Maillard A.; Fiebig M.; Trassin M. Emergence of Ferroelectricity at the Morphotropic Phase Boundary of Ultrathin BiFeO_3_, arXiv 2020.

[ref29] Mundy J. A. Liberating a Hidden Antiferroelectric Phase with Interfacial Electrostatic Engineering. Sci. Adv. 2022, 10.1126/sciadv.abg5860. PMC8809685 35108054

[ref30] Caretta L. Nonvolatile Electric-Field Control of Inversion Symmetry. arXiv, 2022.

[ref31] Levin I. NIST Inorganic Crystal Structure Database (ICSD), 2020.

[ref32] Yang Y.; Ren W.; Stengel M.; Yan X. H.; Bellaiche L. Revisiting Properties of Ferroelectric and Multiferroic Thin Films under Tensile Strain from First Principles. Phys. Rev. Lett. 2012, 109, 057602 10.1103/PhysRevLett.109.057602. 23006208

[ref33] Kozlenko D. P.; Belik A. A.; Belushkin A. V.; Lukin E. V.; Marshall W. G.; Savenko B. N.; Takayama-Muromachi E. Antipolar Phase in Multiferroic BiFeO_3_ at High Pressure. Phys. Rev. B 2011, 84, 094108 10.1103/PhysRevB.84.094108.

[ref34] Lifshitz E. M.; Landau L. D. Statistical Physics; Oxford Butterworth-Heinemann: 1908; Vol. 5, p 544.

[ref35] Deringer V. L.; Caro M. A.; Csányi G. Machine Learning Interatomic Potentials as Emerging Tools for Materials Science. Adv. Mater. 2019, 31, 1902765 10.1002/adma.201902765. 31486179

[ref36] Deringer V. L.; Pickard C. J.; Csányi G. Data-Driven Learning of Total and Local Energies in Elemental Boron. Phys. Rev. Lett. 2018, 120, 156001 10.1103/PhysRevLett.120.156001. 29756876

[ref37] Kresse G.; Hafner J. Ab Initio Molecular Dynamics for Liquid Metals. Phys. Rev. B 1993, 47, 558–561. 10.1103/PhysRevB.47.558. 10004490

[ref38] Kresse G.; Hafner J. Ab Initio Molecular-Dynamics Simulation of the Liquid-Metal–Amorphous-Semiconductor Transition in Germanium. Phys. Rev. B 1994, 49, 14251–14269. 10.1103/PhysRevB.49.14251. 10010505

[ref39] Kresse G.; Furthmüller J. Efficiency of Ab-Initio Total Energy Calculations for Metals and Semiconductors Using a Plane-Wave Basis Set. Comput. Mater. Sci. 1996, 6, 15–50. 10.1016/0927-0256(96)00008-0. 9984901

[ref40] Kresse G.; Furthmüller J. Efficient Iterative Schemes for Ab Initio Total-Energy Calculations Using a Plane-Wave Basis Set. Phys. Rev. B 1996, 54, 11169–11186. 10.1103/PhysRevB.54.11169. 9984901

[ref41] Blöchl P. E. Projector Augmented-Wave Method. Phys. Rev. B 1994, 50, 17953 10.1103/PhysRevB.50.17953. 9976227

[ref42] Kresse G.; Joubert D. From Ultrasoft Pseudopotentials to the Projector Augmented-Wave Method. Phys. Rev. B 1999, 59, 1758–1775. 10.1103/PhysRevB.59.1758.

[ref43] Monkhorst H. J.; Pack J. D. Special Points for Brillouin-Zone Integrations. Phys. Rev. B 1976, 13, 5188–5192. 10.1103/PhysRevB.13.5188.

[ref44] Perdew J. P.; Ruzsinszky A.; Csonka G. I.; Vydrov O. A.; Scuseria G. E.; Constantin L. A.; Zhou X.; Burke K. Restoring the Density-Gradient Expansion for Exchange in Solids and Surfaces. Phys. Rev. Lett. 2008, 100, 136406 10.1103/PhysRevLett.100.136406. 18517979

[ref45] Dudarev S. L.; Botton G. A.; Savrasov S. Y.; Humphreys C. J.; Sutton A. P. Electron-Energy-Loss Spectra and the Structural Stability of Nickel Oxide: An LSDA+U Study. Phys. Rev. B 1998, 57, 1505 10.1103/PhysRevB.57.1505.

[ref46] Chang C.-C.; Lin C.-J. LIBSVM. ACM Transactions on Intelligent Systems and Technology 2011, 2, 1–27. 10.1145/1961189.1961199.

[ref47] Drucker H.; Burges C. J.; Kaufman L.; Smola A.; Vapnik V.; et al. Support Vector Regression Machines. Adv. Neural Inf. Process Syst. 1997, 9, 155–161.

[ref48] Pedregosa F.; et al. Scikit-Learn: Machine Learning in Python. J. Mach. Learn. Res. 2011, 12, 2825–2830.

[ref49] Momma K.; Izumi F. VESTA 3 for Three-Dimensional Visualization of Crystal, Volumetric and Morphology Data. J. Appl. Crystallogr. 2011, 44, 1272–1276. 10.1107/S0021889811038970.

