# Supplementary material for: Physics-Guided Descriptors for Prediction of Structural Polymorphs
Source: J Phys Chem Lett. 2022 Aug 3;13(31):7342–9. doi: 10.1021/acs.jpclett.2c01876 (PMC9376952; doi:10.1021/acs.jpclett.2c01876)
Supplement: Supplementary file 1 — jz2c01876_si_001.pdf [file jz2c01876_si_001.pdf]

Supporting information for "Physics-guided Descriptors for Prediction of Structural Polymorphs"

Bastien F. Grosso,* Nicola A. Spaldin, and Aria Mansouri Tehrani*

Materials Theory, ETH Zürich, Wolfgang-Pauli-Strasse 27, 8093 Zürich, Switzerland

E-mail: b.grosso@ucl.ac.uk; aria.mansouri.t@mat.ethz.ch

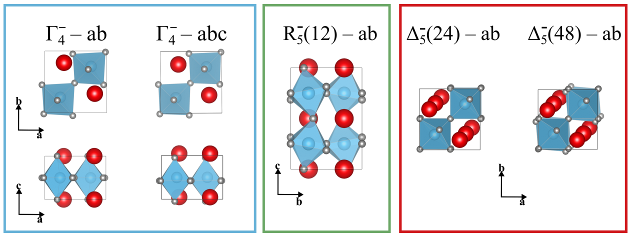

Figure S1: Structures of the five remaining distortion modes, not presented in the main text, with distortion along other orientations than a principal lattice vector. The boxed colours have the same meaning than in Fig. 1.

Table 1: New low-energy phases identified in this work. We give the symmetry (space group), lattice vectors, angle (indicated if different than $90°$) and energy relative to the fully relaxed $R3c$ ground state. The symmetries are determined using Pymatgen[1] with a tolerance of 10^{-3} Å.

	Lattice parameters				Energies
**Symmetry**	a [Å]	b [Å]	c [Å]	Angles [deg.]	Energy [meV/f.u.]
P1	5.54	11.14	15.55	$\alpha = 90.020, \beta = 90.087, \gamma = 90.023$	48
	5.55	11.17	7.76	$\alpha = 90.175, \beta = 90.012$	54
	5.52	5.54	15.84	$\alpha = 90.492, \beta = 90.233, \gamma = 90.061$	57
$P2_1$	5.48	5.57	15.75	$\beta = 90.008$	23
	5.52	11.04	15.68	$\beta = 90.272$	44
	5.55	11.02	15.62	$\beta = 90.085$	54
	5.54	11.15	7.76	$\beta = 90.301$	64
Pm	5.52	15.67	11.10	$\beta = 90.128$	45
	5.53	11.09	7.78	$\beta = 90.006$	56
	5.58	15.49	5.59	$\beta = 90.006$	82
	5.50	15.53	5.56	$\beta = 90.001$	96
Pc	7.77	11.15	5.52	$\beta = 90.001$	69
Cc	7.79	7.81	15.92	$\beta = 90.623$	68
$P2_1/m$	5.52	11.11	15.66	$\beta = 90.269$	46
$P2_1/c$	5.49	11.15	15.67	$\beta = 90.707$	68
$Pmc2_1$	5.55	11.00	7.79		41
	5.53	11.10	15.65		42
$Pmn2_1$	5.53	5.59	7.85		88
$Cmc2_1$	5.62	11.15	7.81		96
Ima2	5.58	5.59	7.75		77
Pbca	5.57	11.04	15.53		55

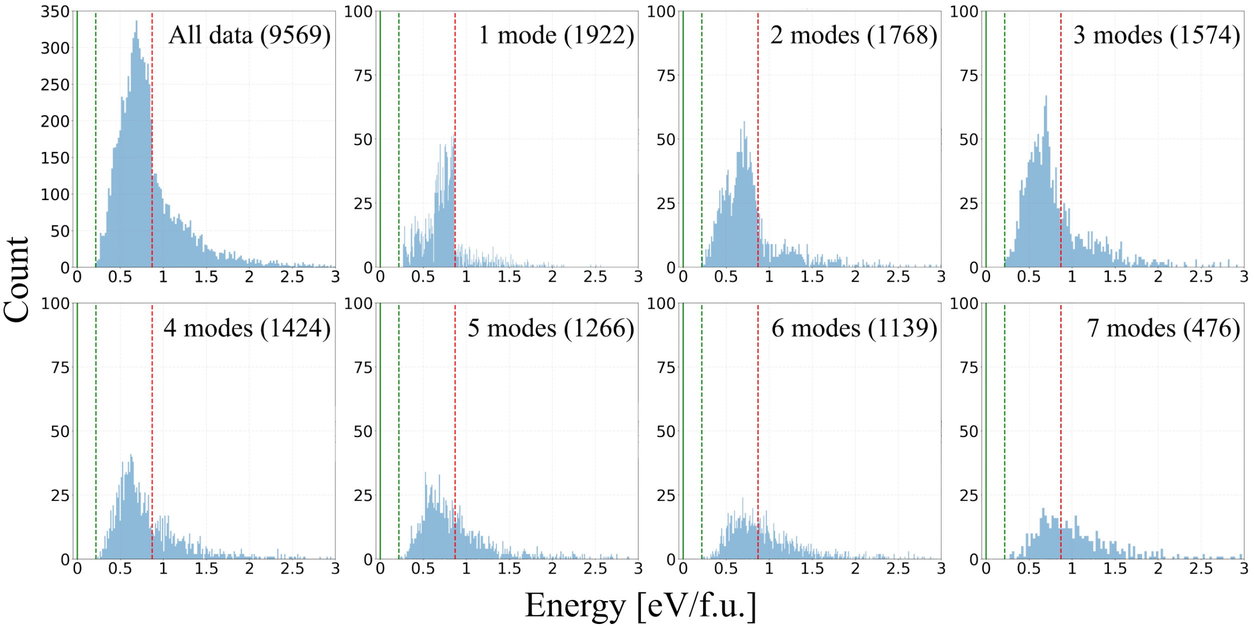

Figure S2: Histograms of the energies of the structures in the training set. The whole training set generated combines 9569 structures (top-left) distributed in structures combining, from top-left to bottom-right, only 1,2,3,4,5,6 and 7 modes, respectively. The number of structures included for each mode is indicated next to the corresponding legend in parenthesis. The continuous green vertical line represents the reference energy of the $R3c$ ground state. The dashed green vertical line shows the energy of the constrained $R3c$ structure where the $R3c$ coordinates have been constrained to the cubic parent structure (volume and angles), and the dashed red vertical line displays the energy of the cubic parent structure (amplitudes of all the modes set to zero). The bins have a width of 10 meV in all plots except the 7 modes one, where the bin size is 30 meV for better visualization.

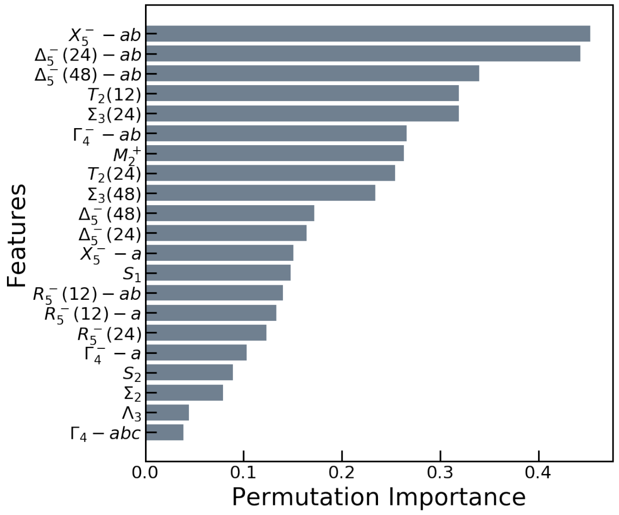

Figure S3: Calculated permutation importance of features of our support vector regression model using 30 repetitions.

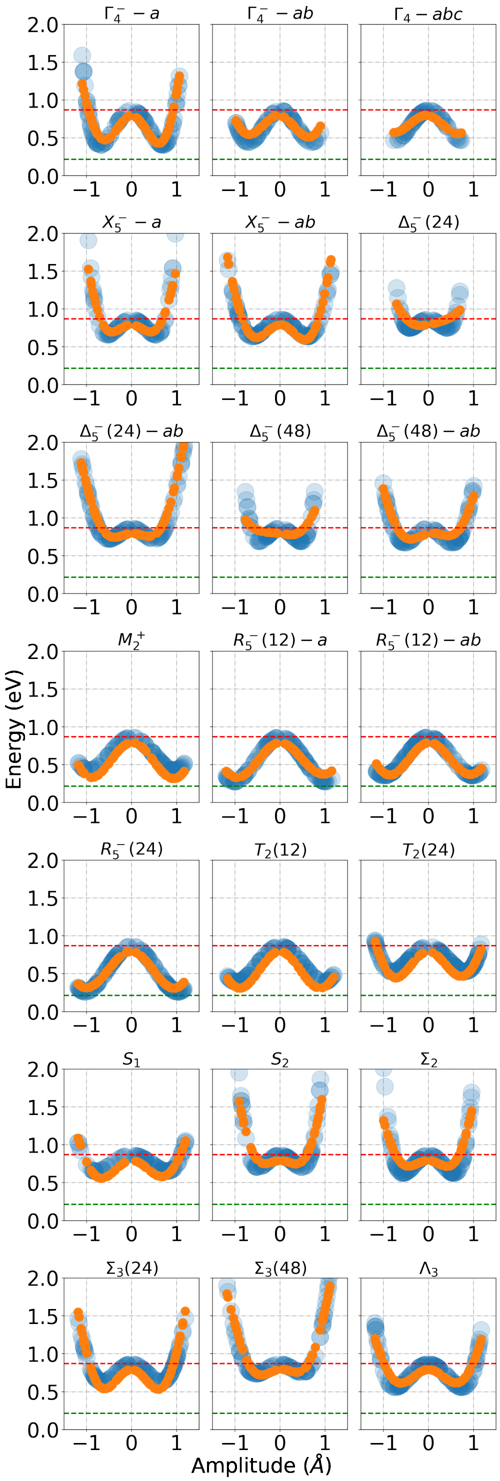

Figure S4: Comparison of the machine-learning predicted variation of the energies as a function of distortion mode amplitude (orange circles) with calculated DFT energies (blue circles).

References

(1) Ong, S. P.; Richards, W. D.; Jain, A.; Hautier, G.; Kocher, M.; Cholia, S.; Gunter, D.; Chevrier, V. L.; Persson, K. A.; Ceder, G. Python Materials Genomics (Pymatgen): A Robust, Open-Source Python Library for Materials Analysis. *Comput. Mater. Sci.* **2013**, *68*, 314–319.
