# Supplementary material for: Physics-Guided Descriptors for Prediction of Structural Polymorphs
Source: J Phys Chem Lett. 2022 Aug 3;13(31):7342–9. doi: 10.1021/acs.jpclett.2c01876 (PMC9376952; doi:10.1021/acs.jpclett.2c01876)
Supplement: Supplementary file 2 — jz2c01876_si_002.pdf [file jz2c01876_si_002.pdf]

jz-2022-01876x.R1

Name: Peer Review Information for "Physics-guided Descriptors for Prediction of Structural Polymorphs"

First Round of Reviewer Comments

Reviewer: 1

Comments to the Author

The work described in this manuscript it timely and important. The authors correctly note that descriptor choice in any ML study is dependent upon the desired outcome that one wishes to predict or model. A one-size-fits-all approach can be burdensome and computational quite expensive, leading to datasets with many uninformative descriptors. The problem of predicting possible structures in systems for which different symmetries and distortions are known is important. While BFO is the subject of this work, the approach could certainly be extended to many structures, extending beyond oxide perovskites. As such, this manuscript is suitable for publication, subject to some minor revisions noted below.

1. The distinction between the content for Figure 1a vs 1b is unclear. Perhaps another box the includes the blue, green, magenta and red boxes could be added.

2. 6 additional linear combinations are shown in Figure 1b, but only 1 is shown. Why? Inclusion of the remaining 5 should be present in the supporting information at the least.

3. The inclusion of a simple filter to remove non-physical structures is appropriate. It appears that only Fe-O distances were included. Is this correct? Analogous cutoff distances for Bi-O distances and even O…O distances (problematic in heavily distorted structures) could certainly be used as well.

Reviewer: 2

Comments to the Author

In this work, the authors introduce a machine learning-based scheme to predict low-energy polymorphs by introducing physics-guided descriptors based on structural

distortion modes. The descriptors are designed by taking care of possible symmetries and distortion modes of different polymorphs. The method is innovative, and the results are solid. My only concern is that it is not obvious to me that this method will be useful for any challenging applications, and hence I am not sure whether it can meet the publication standard of JPCL or not.

In detail, I feel that if possible, to make the paper more convincing and compelling, the authors should give one more example showing: 1) this method can be used to deal with many other similar cases in an efficient way, without the need to do many analyses but simply comparing the results obtained by DFT

and the ML model; or 2) this method can be used to deal with more complicated, but practical, applications. For example, its extension to treating doping, defects, etc.

Author's Response to Peer Review Comments:

Dear Prof. Editor,

Thank you very much for considering our manuscript for potential publication and for sending us the reviews. We are grateful for the constructive feedback and effort to strengthen our manuscript made by the reviewers. We have carefully considered their comments and you can find a point by point answer as well as the parts added to the main text in red.

We also made the non scientific changes and hope that our changes will be well received.

Kind regards,

Dr. Bastien F. Grosso

Response letter for Physics-guided Descriptors for Prediction of Structural Polymorphs

We appreciate the interest of both reviewers in our manuscript and their recognition of our work's **high novelty**, **high scholarly presentation**, and possible **interest to a wider audience** of physical chemists.
Our point-by-point responses to the reviewers' comments are listed below, with our reply written in blue and extracts from the revised text as well as edits and additions to the main text and supplementary information coloured in red.

Reviewer: 1

Recommendation: This paper is publishable subject to minor revisions noted. Further review is not needed.

Comments:
The work described in this manuscript it timely and important. The authors correctly note that descriptor choice in any ML study is dependent upon the desired outcome that one wishes to predict or model. A one-size-fits-all approach can be burdensome and computational quite expensive, leading to datasets with many uninformative descriptors. The problem of predicting possible structures in systems for which different symmetries and distortions are known is important. While BFO is the subject of this work, the approach could certainly be extended to many structures, extending beyond oxide perovskites. As such, this manuscript is suitable for publication, subject to some minor revisions noted below.

We thank the reviewer for their very positive comments and for emphasizing that our approach, while applied to BFO, could be extended to other materials even beyond oxide perovskites. We also appreciate that although considered "as such suitable for publication", the reviewer provided us with a small list of valuable points that has led to increased clarity of our revised manuscript.

1. The distinction between the content for Figure 1a vs 1b is unclear. Perhaps another box the includes the blue, green, magenta and red boxes could be added.

Following the reviewer's suggestion, we have added another box around the coloured boxes in Figure 2 of the revised manuscript for better clarity.

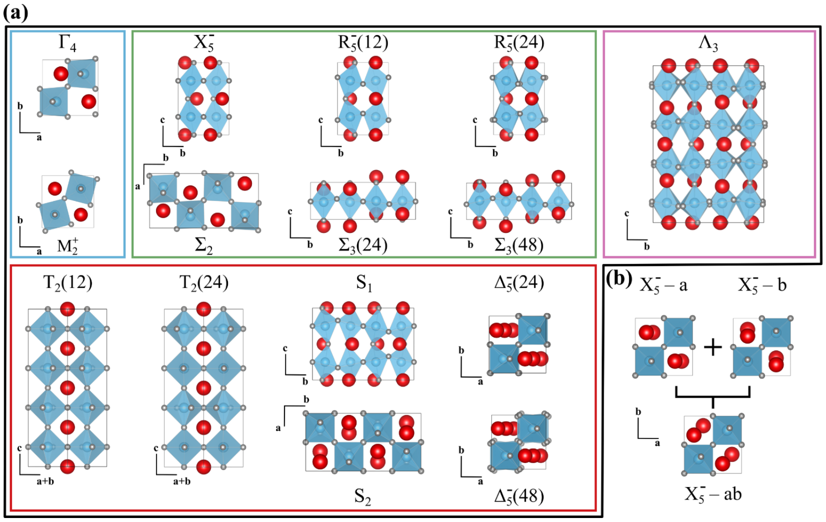

2. 6 additional linear combinations are shown in Figure 1b, but only 1 is shown. Why? Inclusion of the remaining 5 should be present in the supporting information at the least.

We thank the reviewer for this suggestion. We initially did not show the five remaining linear combinations for the sake of space but agree that this is relevant information that we added in the supporting information as Figure S1.

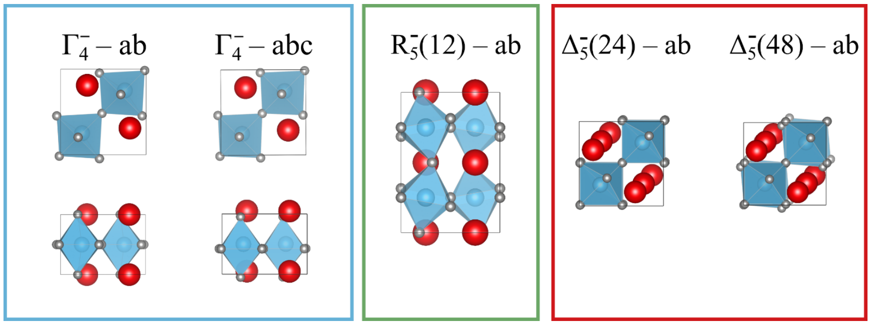

3. The inclusion of a simple filter to remove non-physical structures is appropriate. It appears that only Fe-O distances were included. Is this correct? Analogous cutoff distances for Bi-O distances and even O…O distances (problematic in heavily distorted structures) could certainly be used as well.

We agree with the reviewer, analogous cutoff distances are also important. While we use the Fe-O distance as cutoff, we applied it to all pairs of atoms. Practically, for a given structure we computed the minimum distance between any pair of atoms and discarded the structure if that distance was smaller than our cutoff. We chose the Fe-O distance as cutoff because it is usually the smallest distance between atoms in regular BFO, nevertheless this is defined as a parameter in the codes provided and could easily be adapted to other values for different classes of materials. That been said, we realised that our formulation was confusing and changed it for:

"Based on the distribution of the Fe--O bond lengths in all structures containing these ions in the ICSD database, we keep only structures with the minimum distance between any pair of atoms larger than 1.8 Å. Note that the Fe--O bond lengths were chosen because it is usually the smallest distance in regular BFO."

Additional Questions:
Urgency: High

Significance: High

Novelty: High

Scholarly Presentation: High

Is the paper likely to interest a substantial number of physical chemists, not just specialists working in the authors' area of research?: Yes

----

Reviewer: 2

Recommendation: This paper is probably publishable, but major revision is needed; I do not need to see future revisions.

Comments:
In this work, the authors introduce a machine learning-based scheme to predict low-energy polymorphs by introducing physics-guided descriptors based on structural distortion modes. The descriptors are designed by taking care of possible symmetries and distortion modes of different polymorphs. The method is innovative, and the results are solid.

We thank reviewer 2 for qualifying our method as "innovative" and our results as "solid".

My only concern is that it is not obvious to me that this method will be useful for any challenging applications, and hence I am not sure whether it can meet the publication standard of JPCL or not.

We thank reviewer 2 for this comment pointing to a crucial aspect of our work. According to Web of Science, in the last five years more than 3000 papers containing BiFeO3 in the title have been published, nevertheless only handful of polymorphs were identified until recently and more than half of them have been discovered by our method. Therefore, we are confident to say that we already applied this method to a challenging application. Nevertheless, we think that reviewer 2 made a very significant observation here that made us realise that we did not motivate enough the importance of identifying polymorphs in materials. We integrated reviewer 2 concern by adding the following paragraph in the introduction:

"The advantage of descriptors based only on the chemical composition such as atomic number, covalent radius, number of valence electrons, etc., is that no prior knowledge of the system is

required. However, while this approach has been successful in predicting different properties such as band gap, hardness, and thermodynamic stability, it is not capable of distinguishing between different structures of the same composition (polymorphs). This problem is highly important since, while metastable in bulk, structural polymorphs can in many cases be stabilized through strain, pressure, or electrostatic boundary conditions and often exhibit new properties absent in the bulk ground state enabling new applications. It is, therefore, crucial to incorporate the influence of structural features to correctly describe phenomena such as metal-insulator transitions or ferroelectricity, among others. Descriptors based on structural features, such as the Smooth Overlap of Atomic Positions (SOAP), Minimum bounding ellipsoid (MBE), ordered eigenvalues of the Coulomb matrix, or universal fragment descriptors have been developed in order to capture the local environment of the atoms and encode structural features."

In detail, I feel that if possible, to make the paper more convincing and compelling, the authors should give one more example showing: 1) this method can be used to deal with many other similar cases in an efficient way, without the need to do many analyses but simply comparing the results obtained by DFT and the ML model; or 2) this method can be used to deal with more complicated, but practical, applications. For example, its extension to treating doping, defects, etc.

We thank the reviewer for the suggestions. For suggestion (1), we would like to point out that, even though our method is less computationally expensive than traditional crystal structure prediction methods that are only based on DFT, it still requires generating a set of DFT training data. We also would like to point out that we selected BFO as a phase-space with known and rich structural complexity showing the strength of our method. This has also been observed and pointed out by reviewer 1:" While BFO is the subject of this work, the approach could certainly be extended to many structures, extending beyond oxide perovskites" In addition, reproducing this study on another material would be an entire second paper and probably several months of work.

Therefore, we believe that the reviewer is not asking for a repeat of the whole process on another material but to point out the possible extension of the application of our method to other classes of material. In the revised manuscript, we have now emphasized the application of our method to determining the structural polymorphs of other materials with the example of metallic halide perovskites more clearly. Furthermore, while we initially suggested the possible application of our method in creating interatomic potentials or Landau theory for phase transitions, we added another possible extension of our work in the description of symmetry-allowed combinations of magnetic moments in complex magnetic systems. We are confident that these possible extensions demonstrate the potential of our method further than the study case chosen here.

Finally, we agree with the reviewer that incorporating the effect of doping and defects would be very interesting however is outside of the scope of the current manuscript. Defects induce long-range interactions that are very challenging and expensive to capture using DFT methods as well as incorporating into features suitable for ML. The conclusion reads now:

"Our approach of utilizing distortion modes as building blocks provides a facile way to generate crystal structures and navigate the energy landscape by controlling the coefficients of their amplitudes. Additionally, while we demonstrated the relevance of the use of distortion modes as descriptors through the search for new metastable phases in BFO, our framework can easily be

adapted to other materials, and could be used for example in materials undergoing multiple polymorph transitions such as metallic halide perovskites. Moreover, our method could provide significant insights for many other applications. One example is Landau theory for phase transitions. To elaborate, building a Landau model to study phase transitions in materials involves the study of couplings between distortion modes, which is often limited to second or third order due to the high computational cost. Our model implicitly contains these couplings, which could readily be extracted at minimal computational cost if one would beforehand relax the volume of the structures (in the training set) to avoid the presence of resulting stress contributions to the energy. Another potential application of our methodology is in the field of ML-driven interatomic potentials with a focus on crystal structure prediction. Constructing machine learning-guided interatomic potentials involves creating an efficient training set of structures. While a random sampling of the phase space is a valid strategy and has presented great success in elemental systems, we believe that our approach can be a useful complement to efficiently exploring phase spaces and predicting energetically accessible polymorphs.

Finally, while the building blocks of the modes in our example are the atomic displacements, the use of group/subgroup relations and the corresponding irreducible representations as descriptors in machine learning should be more widely applicable. For example, symmetry-allowed combinations of magnetic moments could be used as a basis to search for low-energy magnetic orderings in complex antiferromagnetic systems."

Additional Questions:
Urgency: Moderate

Significance: Moderate

Novelty: High

Scholarly Presentation: High

Is the paper likely to interest a substantial number of physical chemists, not just specialists working in the authors' area of research?: Yes
